# Collectanea Medica: Consisting of Anecdotes, Facts, Extracts, Illustrations, &c.

**Published:** 1819-09

**Authors:** 


### COLLECTANEA MEDICA:

CONSISTING OF

### ANECDOTES, FACTS, EXTRACTS, ILLUSTRATIONS, &c.

*Relating to the History or the Art of Medicine, and the Auxiliary Sciences.*

---

Floriferis ut apes in saltibus omnia libant,

Omnia nos itidem depascimur aurea dicta.

---

### *Remarks on what takes place in some Animals relative to the Excretion of Milk.*

[BORDEU, *Rechérches sur les Glandes*, § lxxiv.]

THOSE who have often witnessed the milking of cows, or who have had the curiosity to attempt effecting it themselves, must have perceived, that, as the peasants express it, *every body has not a good hand for it.*

Any rude compression of the teat will not do; it should be gently titillated and elongated; we then see the cow fix herself in her position, extend her thighs a little, and then the milk flows in abundance. If compression or extension of the teat were only necessary, every-body could milk an animal.

It often happens that a person who knows very well how to milk cannot get milk from certain cows. Some are delicate in this respect, others are trickish: the former can only give their milk to certain persons; the latter will only do so to those that excite them in a certain manner. They commonly habituate themselves to those who have been accustomed to milk them; and it is often useless for strangers to attempt to effect it.

We sometimes see peasants menace, and even beat, their ewes, cows, and goats, until they choose, as they say, to give their milk; of which they are sometimes very avaricious. Some will only give it when they are amused by having something to eat set before them; others would never give it if their attention were thus distracted: there are in this respect many variations.

What happens with respect to their sucklings, also merits attention. It is known that they seize the teat, and that they elongate it, by pressing it in a sort of canal which they form with their tongue; they suck thus, and labour some time without being able to get any milk, which comes at length when the mother places herself in a certain posture: if she walks about, or if she amuses herself, the suckling does not get much; it is necessary that the mother be occupied by what regards her young one.

When the mother does not give her milk properly, what is it that the young one does? We see it assume a variety of motions; it shakes the teat, and presses it towards the contiguous parts: these agitations make the milk flow, not because they compress the udder, but because they excite it, and put it into action.

A suckling which displeases its mother, as sometimes happens, or a stranger, that the mother will recognize by the rude efforts he makes, and the irritations he excites, will try in vain; he will not get a drop of milk; the mother preserves it for her favourite.

We have been assured by peasants, that there have been vicious cows, which would neither give any milk to their sucklings, nor to those persons who tried to milk them, and which nevertheless appeared to be well adapted for the having of milk. On watching them during the night, it has been seen that serpents came and hung by their teats, and sucked them, which the cows suffered with so much pleasure, that they showed their passion by continued lowings. It is a received opinion amongst the Pyrennees, that serpents have the talent of titillating the cows to such a degree, that, when they have been sucked by these animals, they will not suffer either their sucklings or the peasants to milk them.

There are women who assume an affection for certain children; there are also amongst brute animals some mothers who only take pleasure from certain sucklings: this is commonly their own, as we have already observed, or that to which they have been habituated. But some have been seen to form an attachment to young animals, which they would have fled from, or devoured, if they had not been retained by the delight of the titillation they have endured. Bitches, the most eager after game, have been known to suckle rabbits and squirrels, as well as young wild hogs and wolves, which certainly possessed their natural ferocity; since animals of the same species with the mother who nourished them, have fled from them with terror, or pursued them with rage.

There are some young animals, which, not knowing how to suck, disgust their mothers, whom the peasants are obliged to render favourable to them, by accustoming their teats to the sensation which the suckling, not having the talent to flatter the mother, excites; for the mother will only nourish it on the condition that she shall herself have her share of pleasure.

To these remarks we should add, that, with respect to women, as well as to cows, goats, &c. those which have the largest mammæ are not those which give the most milk. They should be sensible in proportion as they are large, without which they become flabby or loaded with fat: they should also be disposed to enter into contraction, and to take on that action which elicits the milk and causes it to flow.

It must not be forgotten, that, if a mother be not for a certain length of time excited by her suckling, the breasts forget their proper function; they subside, and become indolent, and without action. There are some, however, which can recover it by simple suction; the breasts then awake from their state of drowsiness.

Precocious girls have sometimes been seen, who, having habitually suffered themselves to be sucked, have had milk formed in their breasts; but we know that the breasts commonly only dispose themselves to their functions in consequence of pregnancy. Although it may have happened that men have had milk, as it has been stated, and that this milk has been different from a species of serous fluid with which the breasts of young persons are more or less filled towards the age of puberty, we may dispense with the enquiry after the cause of this rare phenomenon.

Are not these reflections, then, which might be carried to a much greater length, as it would be difficult to explain all the phenomenon relating to this subject; are they not sufficient to authorize the assertion, that the excretion of milk depends, at least in part, as we have already said, on the particular erection of the breasts themselves?

---

### COLLECTION OF RARE AND INTERESTING CASES.

[*Continued from page* 116.]

*Polyphagism.*—All the polyphagists whose wonderful deeds are recorded in history, are superseded by the famous Tarrare, who was known to all Paris, and who died at Versailles about twenty years since, at the age of twenty-six years.

M. le Baron Percy, who saw Tarrare, and who made some investigations respecting this singular personage, has given us the history of him, in a very curious memoir on Polyphagy: it is from this memoir that I shall extract the particulars I am about to relate of Tarrare. Tarrare has renewed amongst us the fable of Erisichton, who, according to Ovid, devoured at one meal what might have sufficed for a whole city, or a whole nation.

*——quod urbibus esse,*

*Quodque satis poterat populo.*

At seventeen years of age, Tarrare weighed only one hundred pounds, and was already able to eat, in twenty-four hours, a quarter of a bullock of that weight. Having left his parents when very young, (he was of the environs of Lyons,) sometimes begging, sometimes stealing, to obtain subsistence, he attached himself to one of the shows on our boulevards, where we see exhibit themselves, in turn, Gill, Harlequin, and Punchinello. One time, on the stage, he defied the public to satiate him, and ate in a few minutes a pannier-full of apples, furnished by one of the spectators; he swallowed flints, corks, and all that was presented to him. At the commencement of the war Tarrare entered into a batallion; he served all the young men in easy circumstances in the company, did all their jobs for them, and ate up the rations they left for him. Famine nevertheless gained upon him; he fell sick, and was taken to the military hospital at Soultz. On the day of his entry he received a quadruple allowance; he devoured the food refused by the other patients, and the scraps about the kitchen; but his hunger could not thus be appeased. He got into the apothecary's room, and ate there the poultices, and everything he could seize. "Let a person imagine," says M. Percy, "all that domestic and wild animals, the most filthy and ravenous, are capable of devouring, and they may form some idea of the appetite, as well as of the wants, of Tarrare." He would eat dogs and cats. One day, in the presence of the chief physician of the army, Dr. Lorence, he seized by the neck and paws a large living cat, tore open its belly with his teeth, sucked its blood, and devoured it, leaving no part of it but the bare skeleton: half an hour afterwards he threw up the hairs of the cat, just as birds of prey, and other carnivorous animals, do. Tarrare liked the flesh of serpents; he managed them familiarly, and ate alive the largest snakes *(couleuvres)* without leaving any part of them. He swallowed a large eel alive without chewing it, but we thought we perceived him crush its head between his teeth. He ate, in a few instants, the dinner prepared for fifteen German labourers: this repast was composed of four bowls of curdled milk, and two enormous hard puddings. After this the belly of Tarrare, commonly lank and wrinkled, was distended like a balloon: he went away, and slept until the next day, and was not incommoded by it. M. Comville, the surgeon-major of the hospital where Tarrare then was, made him swallow a wooden case, enclosing a sheet of white paper: he voided it the following day by the anus, and the paper was uninjured. The general-in-chief had him brought before him; and, after having devoured in his presence nearly thirty pounds of raw liver and lights, Tarrare again swallowed the wooden case, in which was placed a letter to a French officer, who was a prisoner to the enemy. Tarrare set out, was taken, flogged, imprisoned; voided the wooden case, which he had retained thirty hours, and had the address to swallow it again, to conceal the knowledge of its contents from the enemy. They tried to cure him of this insatiable hunger, by the use of acids, preparations of opium, and pills of tobacco; but nothing diminished his appetite and his gluttony. He went about the slaughter-houses and bye-places, to dispute with dogs and wolves the most disgusting aliments. The servants of the hospital surprised him drinking the blood of patients who had been bled, and in the dead-room devouring the bodies. A child fourteen months old disappeared suddenly; fearful suspicions fell on Tarrare; they drove him from the hospital. M. Percy lost sight of him for four years: at the end of this time he saw Tarrare at the civil hospital at Versailles, where he was perishing in a tabid state. This disease had put a stop to his gluttonous appetite. He at length died in a state of consumption, and worn out by a purulent and fetid diarrhœa, which announced a general suppuration of the viscera of the abdominal cavity. His body, as soon as he was dead, became a prey to an horrible corruption. The entrails were putrefied, confounded together, and immersed in pus: the liver was excessively large, void of consistence, and in a putrescent state; the gall-bladder was of considerable magnitude; the stomach, in a lax state, and, having ulcerated patches dispersed about it, covered almost the whole of the abdominal region. The stench of the body was so insupportable, that M. Tessier, chief surgeon of the hospital, could not carry his investigation to any further extent.

Tarrare was of a middle-sized stature; his habit of body was weak and slender; he was not of a ferocious spirit; his look was timid; the little hair he had preserved, although very young, was very fair, and extremely fine. His cheeks were sallow, and furrowed by long and deep wrinkles: on distending them, he could hold in them as many as a dozen eggs or apples. His mouth was very large; he had hardly any lips; he had all his teeth, the molares were much worn away, and the colour of their enamel streaked like marble; the space between the jaws, when they were fully separated, measured about four inches: in this state, with the head inclined backwards, the mouth and œsophagus formed a rectilinear canal, into which a cylinder of a foot in circumference could be introduced without touching the palate. Tarrare, says M. Percy, was constantly covered with sweat, and from his body, always burning hot, a vapour arose, sensible to the sight, and still more so to the smell. He often stank to such a degree, that he could not be endured within the distance of twenty paces. He was subject to a flux from the bowels, and his dejections were fetid beyond all conception. When he had not eaten copiously within a short time, the skin of his belly would wrap almost around his body. When he was well satiated with food, the vapour from his body increased, his cheeks and his eyes became of a vivid red; a brutal somnolence, and a sort of hebitude, came over him while he digested. He was in this state troubled with noisy belchings, and made, in moving his jaw, some motions like those of deglutition. M. Percy never saw in him any signs of rumination. The young Tarrare was almost devoid of force and of ideas. When he had eaten to a moderate extent, and his hunger only appeased, he was quick and active; he was heavy and sleepy only when he had eaten to excess.

---

### *On the Honorary Rewards of a Physician.*

[From the Παραγγελίαι of Hippocrates.]

When you undertake the care of a patient, if you commence with settling what relates to your rewards, you will lead him to suppose that you will not abandon him in the midst of the treatment you may resort to: if you were to act otherwise, he might think that you would neglect him, or not exercise that careful and strict authority which a physician should assume. It is then of importance, in many respects, that you consider what relates to a recompense for your pains. The conduct of a true physician will, however, on all occasions, be guided by liberality and benevolence rather than by severity; and he will not act otherwise in what relates to his demands of pecuniary rewards: he will also be careful to adapt them to the abilities of individuals. He will indeed, on some occasions, attend on patients without requiring them, especially on the poor man and the stranger: he will here have in return a grateful remembrance for his benefits, and will evince his love of human nature as well as of his art.

### Mr. Carmichael's *Synopsis of Venereal Diseases.*

### I. PAPULAR VENEREAL DISEASE.

| *Primary Symptoms.* | *Remedies.* |
|---|---|
| 1. The simple primary ulcer.<br>2. Patchy excoriation, attended with a discharge. | Astringent washes.<br>Antimonials.<br>Sarsaparilla. |
| 3. Gonorrhœa virulenta, | Astringent washes.<br>Antimonials.<br>Terebinthinates. |
| 4. Buboes, | Leeches.<br>Cold applications.<br>Blisters.<br>Poultices. |

| *Secondary Symptoms.* | |
|---|---|
| 1. Papular eruption, preceded by fever, and ending in desquamation. | General blood-letting, to be adopted when indicated by the fever, and proportioned to its degree. |
| 2. Excoriation of the fauces.<br>3. Swelling of the tonsils and glands of the neck.<br>4. Pains in the larger joints, resembling rheumatism. | Antimonials.<br>Sarsaparilla.<br>*Mercury unnecessary in any stage, and highly injurious until the eruption desquamates, the fever is subdued, and the disorder is evidently on the wane; and then an alterative course of antimony and calomel may occasionally accelerate the cure.* |
| 5. Iritis. | *Full mercurial affection of the system until the inflammation is subdued.*<br>Local bleeding.<br>Blistering. |

### II. PUSTULAR VENEREAL DISEASE.

| *Primary Symptoms.* | *Remedies.* |
|---|---|
| 1. The ulcer with elevated edges, without induration. | Astringent washes.<br>Antimonials.<br>Sarsaparilla. |
| 2. Buboes. | The same treatment as for the buboes in No. 1.<br>Excision of the undermined edges of the bubo. |

| *Secondary Symptoms.* | *Remedies.* |
|---|---|
| 1. Eruption of pustules, in general phlyzacious, preceded by fever, and terminating in ulcers covered with thin crusts that heal from their margins, and, when the disease is on the wane, the eruption desquamates into scaly red blotches. | General blood-letting, as in No. 1.<br>Antimonials.<br>Sarsaparilla.<br>Guaiacum.<br>Tar ointment.<br>Baths of sulphurated kali.<br>Nitro-muriatic acid baths.<br>*Mercury is decidedly pernicious until the pustules terminate in scaly blotches instead of forming ulcers; and then mercurials, in alterative doses, conjoined with sarsaparilla or guaiacum, may occasionally be employed with benefit.* |
| 2. Ulcers on different parts; fauces, in general of a white apthous appearance. | The same general treatment, conjoined with the following local applications:<br>Common detergent gargles.<br>Oxymel Æruginis.<br>Fumigations of factitious cinnabar. |
| 3. Pains in the joints. | The same general treatment, conjoined with the following local applications:<br>Leeches.<br>Fomentations.<br>Bread and water poultices.<br>Blisters.<br>Ointment of tartarised antimony.<br>*Mercury is to be particularly avoided while inflammation of the knee exists.* |

| | |
|---|---|
| 4. Nodes. | The same general treatment, conjoined with the following applications: Leeches. Poultices. Blisters. Ointment of tartarized antimony. Division of the periosteum. *If the preceding remedies prove inefficient, a mercurial course may be of advantage, provided the general disease is on the wane.* |

III. PHAGEDENIC VENEREAL DISEASE.

| *Primary Symptoms.* | *Remedies.* |
|---|---|
| 1. The phagedenic ulcer, 2. The sloughing ulcer. | Bread and water poultices, with opium occasionally added. Warm fomentations. General blood letting, proportioned to the acuteness of pain, inflammation, and symptomatic fever. Antimonials in nauseating doses. Cicuta in full doses. Sarsaparilla. |
| 3. Buboes. | The same treatment as for buboes in No. II. |

*Secondary Symptoms.*

| | |
|---|---|
| 1. Eruption of tubercles, or spots of a pustular tendency, or both intermixed, preceded by fever, and terminating in ulcers covered with thick crusts which often assume a conical form, healing from their centre, and extending with a phagedenic margin. When the disease is on the wane, they desquamate into scaly red blotches. | General blood-letting, as in No. I. II. Antimonials. Sarsaparilla. Guaiacum. Compound powder of ipecacuanha. Arseniate of kali. Nitrous acid. Nitro muriatic acid bath. *Mercury, with the exceptions noticed below, increases the ravages of the disease in all its stages, but the last: it may then be given with safety and advantage, as in No. II.* |
| 2. Ulcers on different parts of the fauces, and particularly the back of the pharynx, frequently engaging the entire fauces, and sometimes extending to the nares and larynx. | The same general treatment, conjoined with the following applications: Oxymel Æruginis. Strong solution of muriate of mercury, or nitrate of silver. Fumigations of factitious cinnabar. *If these should prove inefficient, mercury may be used largely with advantage in checking the progress of the ulceration, even though it should exasperate the general disease.* |
| 3. Ulceration, and caries of the bones of the nose. | The same general treatment, with the yellow and black mercurial washes frequently injected into the nostrils. *If these remedies fail, mercury may be used as above.* |
| 4. Pains of the joints. | The same treatment as in No. II. for the same symptom. |
| Nodes. | The same treatment as in No. II. |

IV. SCALY VENEREAL DISEASE.

| *Primary Symptoms.* | *Remedies.* |
|---|---|
| 1. The chancre, or callous ulcer. 2. Buboes. | *Full courses of mercury for both primary and constitutional symptoms.* |

*Secondary Symptoms.*

| | |
|---|---|
| 1. Eruption of scaly blotches, presenting either the character of lepra or psoriasis, and unattended with any obvious degree of fever. 2. Excavated ulcers of the tonsils. 3. Pains in the joints, tibiæ, cranium, &c. 4. Nodes. | The same local remedies as prescribed for the several symptoms in No. II. N. B. It is yet doubtful whether sarsaparilla and guaiacum may not be substituted for mercury in this as well as the preceding diseases. |

### *Observations relative to the Germination of Vegetables.*

[From the *Inductions Morales et Physiologiques* de M. Keratry, p. 112.]

In the month of September, 1815, walking over my land, where persons were employed digging potatoes *(solanum tuberosum,)* I perceived on the ground a fragment of one, of considerable size, (since it formed the half of a spheroid which would have equalled in bulk a hen's egg,) which I was surprised to see had not furnished any germ or sprout. A superficial examination led me to believe that it had been *eyed* (the eyes, or origin of the germs, removed) before it had been employed as seed, for its exterior was in other respects very healthy in appearance; but what I afterwards discovered with surprise, after I had cleansed it from the earth adhering to it, was, that on the plane of the section there was formed a dozen small tubercles, similar in size to large pins'-heads, the most prominent of which occupied the middle of a curved line, in the manner of a diadem; whilst the others, in their decreasing progression, were ranged towards the extremities of the arch, none of which touched, by any point, the rind which formed the lateral boundaries. I omit nothing; I do not here alter any one of the circumstances; for they all seem to me to be very important.

The little tubercles, furnished with a pre-eminent germ, visible without the aid of the glass, were perfectly formed, touched each other, and made one continuous substance with the pulp that remained solid. I should have expected that this would have undergone the process of fermentation common to farinaceous or mucilaginous substances, destined to nourish the products of vegetation.

Doctor Dubosq, and the "directeur du cadastre," Dessaux, both versed in the knowledge of natural history, and settled in Finistere, have held in their hands this fragment of potatoe, which appeared to them to merit their attention.

Here then is a plant perpetuated without the aid of root, sucker, slip, eye, tuberculated bark, or the bark of branches, often the depositary of germs; and without any development of leaves or of flowers!

Does it not follow from this fact, the truth of which I affirm, that the sappic juice, elaborated in the organs of a vegetable, transmitted by appropriate canals, and perfected in the cellular tissue, after having passed through a proper degree of fermentation, may be fitted to class its molecules according to a regular system of organization? We are even led to believe that it may contain a sufficient quantity of parts proper for the formation of the germ, which would best develope themselves, and unite themselves most easily, by the usual means of fructification, but which, in certain favourable circumstances, may become, in an almost immediate manner, organic wholes.

